# A Systematical Review on ART Use in HTLV Infection: Clinical, Virological, and Immunological Outcomes

**DOI:** 10.3390/pathogens13090721

**Published:** 2024-08-27

**Authors:** Tatiana Fernandez, Cleyde Marconi, Iris Montaño-Castellón, Felice Deminco, Carlos Brites

**Affiliations:** 1 Laboratório de Pesquisa em Infectologia (LAPI), Hospital Universitário Professor Edgard Santos, Federal University of Bahia, Salvador 40110-060, Brazil; tatiana.fernandez@ufba.br (T.F.); marconi.cleyde@gmail.com (C.M.); iris.med.umss@gmail.com (I.M.-C.); fdeminco@gmail.com (F.D.); 2 Programa de Pós Graduação em Medicina e Saúde (PPgMS), Federal University of Bahia, Salvador 40110-060, Brazil

**Keywords:** HTLV, antiretroviral, treatment

## Abstract

Human T-cell lymphotropic virus (HTLV) infection affects over ten million people worldwide, but there is no effective treatment so far. This review describes the virological, immunological, and clinical outcomes of antiretroviral therapy (ART) in people with HTLV infection. This systematic review followed PRISMA reporting guidelines and was registered in PROSPERO: CRD42022350076. The Newcastle–Ottawa Scale, adapted for cross-sectional studies, and Rob-2 were used to assess the methodological quality of these studies. Systematic searches were conducted in the Medline (PubMed), Scopus (Elsevier), Cochrane Library, and Web of Science (Clarivate Analytics) databases. We retrieved data from eight methodologically diverse articles on treatment of patients infected by HTLV-1 or HTLV-2 alone, or coinfected by HIV-1, who received Raltegravir, Tenofovir, Lamivudine, or Zidovudine. The proviral load decreased in three out of seven studies over 4 to 48 weeks of antiretroviral use. Cellular immune response (CD4, CD8, CD25, CD69, and CD71 cells) was evaluated in six studies. While no significant clinical improvement was observed, all studies reported clinical stability during treatment. Despite the demonstrated antiviral activity of ART, in vitro, clinical improvement was not proven. Most studies showed disease stability during ART use, suggesting potential clinical benefits. There is a need of larger, well-controlled trials to define the role of ART in the treatment of HTLV infection.

## 1. Introduction

The human T-cell lymphotropic virus (HTLV) is the causative agent of several diseases, like HTLV-associated myelopathy (HAM/TSP), adult T-cell leukemia/lymphoma (ATLL), and infective dermatitis, among others [1]. It can be transmitted by blood products, by sexual contact, or from mother to child, sharing transmission routes with HIV [1,2,3]. It is estimated that around 5 to 20 million people are infected worldwide [2]. Approximately 10% of the population living with HTLV-1 will develop a clinical disease like HAM/TSP or ATLL [3]. However, there is currently no available treatment to control viral replication or prevent transmission. Drugs capable of reducing PVL could be an important tool to decrease the incidence of HTLV-associated diseases and also impact transmission of the virus.

HTLV-1 infects CD4+ and CD8+ T lymphocytes, with a preferential tropism for CD4+ T lymphocytes. HTLV replication is characterized by the clonal expansion of infected cells and minimal detection of viral particles in plasma [4]. Replicative activities are modulated by regulatory proteins capable of inducing viral transcription and interfering with host cells’ replicative and repair mechanisms. Cells infected by HTLV-1 or HTLV-1/HIV usually present with increased numbers of dysfunctional CD4 T-cells, which can lead to an underestimation of immunodeficiency in HIV–HTLV coinfected patients [5,6]. Therefore, early antiretroviral therapy (ART) is recommended for coinfected patients, even if the CD4 count seems to be in the normal range [7,8].

High HTLV proviral load (PVL) is associated with disease progression [9,10,11]. Some previous studies have shown higher PVL in patients with ATL or HAM/TSP in comparison with asymptomatic carriers [9,10,11]. In addition, comorbidities and parasitic infections (like tuberculosis and *S. stercoralis*) can trigger lymphocytic clonal expansion and increase PVL, consequently. However, there is no established reference value for PVL as a predictor of the development of clinical manifestations associated with HTLV infection [9].

Although HTLV was the first retrovirus to be isolated [11], this infection is considered neglected and still lacks any effective treatment [3]. However, following the success of antiretroviral therapy (ART) against HIV infection, which targets the replication enzymes present in human retrovirus (proteases, reverse transcriptase, and integrase), some studies evaluating the impacts of antiretroviral use on HTLV infection have been conducted, but the results are still controversial. The use of antiretroviral drugs, if effective, could reduce the PVL and the clinical evolution of infection with diseases like HAM/TSP or ATL. This review aims to describe the results of published studies on the use of ART in HTLV infection and its impact on HTLV PVL, as well as clinical and immunological outcomes. Papers on the use of antiretroviral drugs for ATLL treatment were excluded.

## 2. Materials and Methods

### 2.1. Information Sources and Search Strategy

This systematic review was registered at the International Prospective Register of Systematic Reviews (PROSPERO) (registration no. PROSPERO 2022, CRD42022350076).

A comprehensive literature search was performed using the following electronic databases: Medline (PubMed) (229), Scopus (Elsevier) (97), Cochrane Library ()], and Web of Science (Clarivate Analytics) (166). The search included a review of references cited by papers and was finalized on 20 July 2022. One article was retrieved from additional literature. No restrictions were placed on the year or location of publication. The search strategies used to obtain the articles are outlined in Table 1.

### 2.2. Eligibility Criteria

In vivo studies on ART use for treatment of people infected by HTLV, regardless of gender or ethnicity, were eligible.

### 2.3. Exclusion Criteria

Studies on the treatment of individuals with ATLL or with coinfection by other viruses than HIV-1 were excluded. In addition, in vitro studies were not eligible to enter this review.

### 2.4. Main Outcomes

Primary Outcome: Changes in HTLV PVL during antiretroviral treatment.

Secondary outcomes: Clinical changes, CD4+ and CD8+ cell count, CD4+/CD8+ T-cell ratio, mortality.

### 2.5. Assessment of Risk of Bias in Included Studies

The risk of bias in this study was assessed by using the NOS scale, the NOS scale adaptation for cross-sectional studies, and Rob-2 for clinical trials. The mean final scores were defined as moderate or high risk (clinical trial), 6.8 (cohort), and 7 (cross-sectional).

### 2.6. Selection of Studies

**Figure 1:**
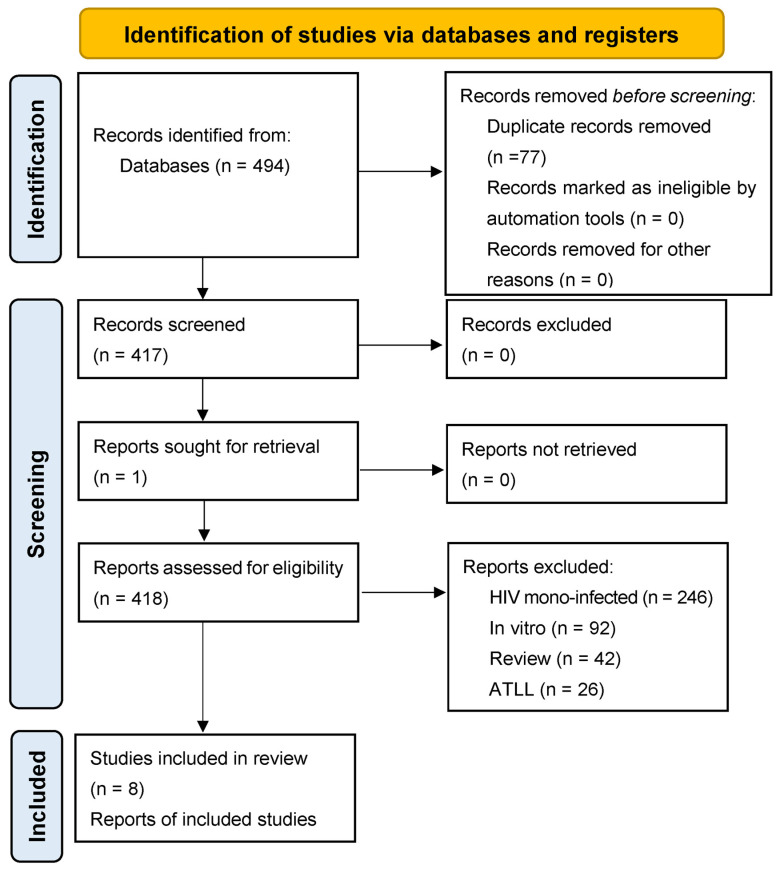
PRISMA 2020 structured search strategy flow diagram.

After using the search strategy described in the Methods section, we identified a total of 494 articles. Seventy-seven duplicates were removed, and retrieval was not possible for one article. A total of 417 articles were eligible for title and abstract reading. By using PICO criteria, 19 articles were selected for full-text reading. Eleven additional full-text articles were excluded because they did not fulfill the eligible criteria. Eight articles met the criteria for inclusion in this systematic review, as shown in Figure 1. The selected articles included 2 clinical trials, 4 cohorts, 1 cross-sectional study, and 1 pilot study.

### 2.7. Data Extraction and Management

This review was conducted in accordance with the “Preferred Reporting Items for Systematic Reviews and Meta-analyzes” protocol (PRISMA, http://prisma-statement.org, access date: 23 April 2024).

The Rayyan Intelligent Systematic Review (https://www.rayyan.ai/) software was used during the screening process. Three reviewers selected articles for inclusion in a blinded fashion. The full texts of any potentially eligible articles were then retrieved and independently assessed for inclusion/exclusion criteria. Disagreements between the reviewers on the eligibility of any study were resolved through discussion with a fourth reviewer. The risk of bias of all included studies was assessed with the Newcastle–Ottawa Scale (NOS) and Rob2.0 for analysis of clinical trial studies. Table 2 summarizes the studies included in this review.

## 3. Results

In this review, we present the data retrieved from eight articles [12,13,14,15,16,17,18,19]. The population of the selected studies was very diverse. Five were HTLV-1 or HTLV-2 monoinfected patients. Two studies included HIV/HTLV-2 coinfected patients. One study used HTLV-1 monoinfected and HIV/HTLV-1 coinfected patients. Other characteristics of the studies’ population are available in Table 2.

### 3.1. Antiretroviral Therapy

Most of the selected articles used only one drug: Raltegravir (RAL) [17,18,19], Tenofovir (TDF) [16], or Lamivudine (3TC) [12]. Taylor et al. [14] (2006), in their placebo-controlled study, used the combination of Zidovudina (AZT) and 3TC. In two studies, the protocol did not provide data about the ART used, which was at the discretion of the assistant physician [13,15].

### 3.2. Proviral Load

The quantification of HTLV proviral load (PVL) in the use of ART was analyzed in seven studies [12,14,15,16,17,18,19]; Zehender et al. [13] (2002) described PVL in only one patient who developed myelopathy, and no significant changes were observed during follow-up. In another study, Taylor et al. [12] (1999) observed a decrease in PVL in patients who received 3TC (median decrease of 1.1 log10), with a PVL nadir occurring between 4 and 24 weeks. Three studies showed no association between PVL changes and antiretroviral use [14,15,16]. In a study by Treviño et al. [17] (2012), a transient decline in PVL was observed in the first 6 months of RAL use in patients with HAM/TSP, followed by a return to baseline values after interruption of therapy. In addition, Enoose-Akahata et al. [19] (2021) did not observe significant changes in proviral load with the use of RAL, but when they performed a subgroup analysis, eight participants with HAM/TSP showed reductions in PVL in PBMC and five participants with HAM/TSP had reductions in PVL in the CSF after 6 months of ART use. On the other hand, Abad-Fernández et al. [18] (2014) carried out a generalized estimating equation model for PVL in relation to time and observed a significant decline in PVL in the period of 24 to 48 weeks in those who received RAL.

### 3.3. Immunological Outcomes

The immunological statuses of patients were analyzed in six of the eight articles [13,14,15,16,18,19]. Taylor et al. [12] (1999) performed analysis of lymphocyte quantification, half-life, and phenotypes in only one patient. Treviño et al. [17] (2012) did not evaluate immunological status. Zehender et al. [13] (2002) identified a lower CD4+ cell count in patients coinfected with HIV/HTLV-2 than that observed in patients infected only by HIV-1. Enoose-Akahata et al. [19] (2021) and Taylor et al. [14] (2006) also observed that individuals on ART tended to have lower lymphocyte counts. On the other hand, Macchi et al. [16] (2011) observed an increase in lymphocyte counts following the use of ART, caused by an increase in both CD4+ and CD8+ cell count. Abad-Fernández et al. [18] (2014) found no significant variation in CD4+ or CD8+ cells in the placebo or in the ART group. One study evaluated the CD4+/CD8+ cell ratio and did not detect any statistical difference between groups [19].

The study that investigated the CD69 and CD71 markers did not observe changes in the frequency of such markers after antiretroviral treatment [14]. As for studies on CD25, the results were divergent: one recorded no post-treatment changes [14], while the other indicated a reduction in this cell’s subpopulation [19].

### 3.4. Clinical Outcomes

Clinical outcomes were evaluated in six articles [12,13,14,16,17,19]. Zehender et al. [13] (2002) observed that the development of peripheral polyneuropathy (PN) was greater in HIV/HTLV-2 coinfected patients and that the use of HAART promoted a reduction in PN incidence. Taylor et al. [12] (1999) detected a clinical improvement in a patient with HAM/TSP that experienced a decrease in PVL during the use of 3TC. Macchi et al. [16] (2011) demonstrated that participants who received TDF for a longer time had improvement in pain and/or gait. Finally, the study by Enose-Akahata et al. [19] (2021) concluded that patients experienced subjective improvement with RAL use, but no significant objective improvements were observed.

## 4. Discussion

In our review, we identified that only two classes of antiretrovirals have been tested in humans: nucleoside reverse transcriptase inhibitors and integrase inhibitors. AZT was the first antiretroviral drug used in HTLV infection [20] and is the only one currently used in clinical practice, as an adjuvant treatment of adult T-cell leukemia. Although there is no consistent evidence to support its use in patients with HTLV infection, in 2013, a case report was published on a HIV/HTLV coinfected patient diagnosed with HAM/TSP and who experienced progressive clinical improvement after using combined therapy (AZT + 3TC) [21]. However, most studies using AZT + 3TC or AZT monotherapy in HAM/TSP included a small sample of participants.

In vitro studies showed that HTLV-1 is highly resistant to 3TC [22,23]. Therefore, the use of this antiretroviral to control HTLV replication has been discouraged, despite conflicting results in the literature [22,23,24]. In our review, Taylor et al. [12] (1999) used 3TC as monotherapy and demonstrated a decrease in PVL in relation to baseline levels, despite subsequent oscillation in the participants’ PVL, probably due to cellular clonal expansion. TDF is another drug, already tested in vitro, that has been shown to inhibit HTLV-1 infection (EC50 = 17.78 ± 7.16 nM) [25]. In vitro, AZT and TDF appear to be the most potent nucleoside reverse transcriptase inhibitors against HTLV (TDF: IC50 5.4 nmol/L versus AZT: IC50 0.11 µmol/L) [24]. However, in this review, the only study on the use of TDF showed neither significant reduction in PVL nor clinical improvement [16].

Currently, integrase inhibitors are considered the first line of treatment for HIV because they are safe and effective. Few studies on the use of integrase inhibitors in HTLV infection have been published to date. An in vitro study demonstrated that RAL could inhibit HTLV transmission in both cell-free and cell-to-cell mechanisms [26]. Another in vitro study described that second-generation integrase inhibitors and Elvitegravir were superior in inhibiting HTLV when compared to RAL [25]. Despite this, to date, only RAL has been studied in human beings, with still-conflicting results, which can be justified by the reduced number of participants and short follow-up time [17,18,19]. Dolutegravir (DTG) is the most used integrase inhibitor in the world; however, there is no published study on the use of DTG for treatment of HTLV infection to date.

Only three out the seven studies analyzed detected PVL decreases, which occurred between 4 and 48 weeks of ART use [14,17,18], and an association between presence of symptoms and higher PVL. Beilke et al. [15] (2007) attributes the PVL increase in patients coinfected by HIV-1 to the immune reconstitution mediated by ART use. However, four studies related here [12,16,17,19] reported PVL variability over time independent of ART use, a fact already detected in other studies that followed HTLV-1 individuals off ART [27,28]. Although some researchers considered the PVL increase in HTLV-1 as one of the main risk factors for symptomatic patients, it seems that PVL alone cannot predict the progression of the disease, which depends on other factors involved in modulation of the immune response, like the increase in Tax expression and the imbalance between pro- and anti-inflammatory cytokines (IFN-γ and IL-10) [29,30]. Nevertheless, it is likely that proviral load, in association with other factors, can be a predictor of HTLV-1/2 [31].

The CD4+ cell count in HIV-1/HTLV-2-coinfected participants studied by Zehender et al. [13] (2002) tended to be lower compared with HIV-1 monoinfected participants. This is in accordance with the findings of Beilke et al. [15] (2007) showing that the CD4+ cell counts of HIV-1/HTLV-1 patients tended to be higher than those with HIV-1/HTLV-2. The target cells of HTLV-2 infection are TCD8+ lymphocytes, which are naturally capable of modulating an immune response to HIV-1 infection and have a higher inhibitory effect on HIV-1 in individuals coinfected by HIV-HTLV-2 than in HIV-1 monoinfected ones [32]. However, these findings were not reported among the articles included in this review that worked with HTLV-2. Abad-Fernández et al. [18] (2014) were also unable to identify a clone expansion in the CD4+ and CD8+ cells of HIV-1/HTLV-2 participants in their results. No explanation was given for this finding by the authors.

Taylor et al. [14] (2006) reported an increase in CD4+ cells in their study groups regardless of the use of combined therapy of AZT and 3TC. Similarly, Macchi et al. [16] (2011) reported increases in both CD4 and CD8 in patients using TDF. Although antiretrovirals can elicit immune reconstitution, there is still no explanation on the action of these drugs in monoinfected HTLV-1 patients, nor why the number of these lymphocytes increases or decreases in some groups of patients.

Enose-Akahata [19] (2021) reported that the frequency of TCD4+/CD25+ cells was much higher in PBMC and cerebrospinal fluid samples from patients with HAM/TSP before the start of RAL therapy compared with the same samples collected from healthy participants. However, 15 months after the start of therapy, the frequency of these cells decreased in the patients with HAM/TSP, with the same drop seen in TCD8+/CD25+ cells, although only in peripheral blood samples. Other cell profiles were also analyzed, such as T-memory lymphocytes and natural killer populations, but without significant changes. CD4+/CD25+ cells are the main reservoirs of the virus, and, as they also show high levels of Tax mRNA in the CSF of patients with HAM-TSP, they may be the target of investigations into their possible role as a biomarker of response to antiretroviral drugs [33,34].

In this review, seven studies showed clinical-related results, mainly focused on neurological manifestations such as HAM/TSP, bladder dysfunction, polyneuropathy, functional impairment, and gait. All showed no worsening (clinical stability) during the treatment and follow-up, most of the studies included patients with more than 8 years since the diagnosis of HTLV, the follow-up time ranged from 6 months to 10 years, and the n of patients was from 4 to 90 patients. The study with the largest number of patients and the longest follow-up [12] showed a transient improvement in one patient during proviral load reduction. No studies reported clinical decline during treatment.

Although the articles included in this review did not show significant clinical improvement, some of them attributed the possibility of no improvement to irreversible nerve damage caused in patients with long-term medical histories [14]. Chronic neuronal dysfunction, both in the central and peripheral nervous systems, leads to cell death and consequent irreversible damage to the nervous system. In the case of infections with known neurotropism, the immune response against infected cells can cause the release of inflammatory cytokines and viral proteins that indirectly damage nervous system cells [35]. However, the great heterogeneity of methodologies used in the studies included in this review, their small sample size, and the short follow-up clearly limit the consistency of their main findings.

The improvement in antiretroviral treatment was followed by a significant decrease in the incidence rates of dementia, vacuolar myelopathy, polyneuropathy, and myositis in people living with HIV [35,36]. By analogy, a population of interest for new studies on ART use in HTLV infection are patients with recent diagnosis and/or oligosymptomatic patients, given that all studies demonstrated the non-progression of disability and/or clinical stability during the use of antiretrovirals. Early initiation of antiretroviral therapy could prevent the long-term neuronal damage caused by the infection, minimizing symptoms already present and preventing their progression.

## 5. Conclusions

Although it has been demonstrated that the use of antiretrovirals inhibits HTLV replication in vitro, the results are still conflicting in clinical practice. Few studies have shown clear improvements in clinical manifestations, and the small sample sizes and methodological differences between the existing trials limit the understanding of the relevance of ART to the treatment of HTLV infection. However, an important finding could be the non-progression of symptoms during the use of antiretrovirals, which was shown in most published studies. Larger, well-controlled trials, with longer follow-up times, are needed to demonstrate the real role of ART in the treatment of HTLV infection.

## Figures and Tables

**Table 1:** Databases accessed with corresponding search strategies.

	Database (Company)	Keyword (MeSH) Terms and Text Word Search
1	Medline (PubMed)	(“Human T-lymphotropic virus 1”[Mesh] OR “HTLV-I Infections”[Mesh] OR “HTLV-II Infections”[Mesh] OR “HTLV-1”[tiab] OR “HTLV-2”[tiab] OR “HTLV-I”[tiab] OR “HTLV-II”[tiab] OR “HTLV”[tiab]) AND (“Anti-Retroviral Agents” OR “dolutegravir” OR “raltegravir” OR “Isentress” OR “elvitegravir” OR “bictegravir” OR “zidovudine” OR “efavirenz”) NOT (“ATL”)
2	Cochrane Library	(“HTLV” OR “ Human T-lymphotropic virus 1” OR “HTLV-I Infections” OR “HTLV-II Infections” OR “HTLV-1” OR “HTLV-2” OR “HTLV-I” OR “HTLV-II”) AND (“Anti-HIV Agents” OR ‘HIV Protease inhibitors” OR “HIV Integrase Inhibitors” OR “Anti-Retroviral Agents” OR “HIV/drug effects” OR “”Drug Ressistance”) NOT (“ATL”)
3	Scopus (Elsevier)	((ALL (“HTLV” OR “ Human T-lymphotropic virus 1” OR “HTLV-I Infections” OR “HTLV-II Infections” OR “HTLV-1” OR “HTLV-2” OR “HTLV-I” OR “HTLV-II”)) AND (ALL (“Paraparesis, Tropical Spastic” OR “HAM/TSP” OR “Paraparesis, Tropical Spastic/therapy”) OR TITLE-ABS-KEY (“Paraparesis, Tropical Spastic” OR “HAM/TSP”))) AND (ALL (“Anti-HIV Agents” OR “dolutegravir” OR “raltegravir” OR “Anti-Retroviral Agents” OR “elvitegravir” OR “bictegravir” OR “zidovudine” OR “efavirenz”) NOT ALL(“ATL”))
4	Web of Science (Clarivate Analytics)	ALL = (“HTLV” OR “ Human T-lymphotropic virus 1” OR “HTLV-I Infections” OR “HTLV-II Infections” OR “HTLV-1” OR “HTLV-2” OR “HTLV-I” OR “HTLV-II”) AND ALL = (“Anti-Retroviral Agents” OR “dolutegravir” OR “raltegravir” OR “Isentress” OR “elvitegravir” OR “bictegravir” OR “zidovudine” OR “efavirenz”) NOT ALL = (“ATL”)

**Table 2:** Summary of studies included in this review.

Study	Design, Country	Population Characteristic	Infection	ART Use	Outcomes
Taylor et al., 1999 [12]	Cohort, United Kingdom	N = 5; female: 4 (80%); mean age: 46.8	HTLV-1 with HAM/TSP: 5 (100%)	3TC (150 mg bid *) (* A patient who had a recent diagnosis of HAM/TSP received AZT (300 mg bid) for 3 months and then switched to 3TC).	HTLV-PVL was reduced in all patients. Clinical improvement was only observed in one patient with recent-onset HAM/TSP during the use of lamivudine, as well as PVL decrease.
Zehender et al, 2002 [13]	Cohort, Italy	N = 90; male: 70%; mean age HIV group: 32 (26–50) mean age HIV/HTLV-2 group: 33 (23–55).	HIV/HTLV-2: 30 (33.3%) HIV: 60 (66.6%)	It was not controlled by the study protocol.	There was no difference between the monoinfected and coinfected groups in mortality or CD4+ cell count. HLTV-2 infection was an independent predictor for developing PN. The incidence of PN decreased during ART use.
Taylor et al., 2006 [14]	RCT, United Kingdom	N = 16, male: 5 (31%); mean age: 57.4	HTLV-1 with HAM/TSP: 16 (100%)	AZT + 3TC (300 mg + 150 mg bid)	There was a trend of decreasing CD8+ cell count with the use of ART. There was no significant change in PVL nor CD4+ cell count. There were no significant changes in pain score, urinary frequency, or nocturia.
Beilke et al., 2007 [15]	Cross-sectional, USA	N = 72, male: 59 (76%) Age: >45 years (72%)	HIV/HTLV-1: 20 (27.7%) HIV/HTLV-2: 52 (72.3%)	Any triple ART	PVL was higher in HIV/HTLV-1 than in HIV/HTLV-2 and in cases with positive PBMC cultures.
Macchi et al., 2011 [16]	Cohort, United Kingdom/Italy	N = 6, Female: 4 (67%); mean age: 44.8 (±15)	HTLV-1 with HAM/TSP: 5 (83.3%); 1 patient with encephalitis (16.7%)	TDF (300 mg qd)	There were increases in CD4+ and CD8+ cell count. No significant clinical improvement was seen, except in those who received TDF for a longer period who experienced improvement in pain and gait. There was no significant change in PVL.
Treviño et al., 2012 [17]	Pilot study, Spain	N = 5; Female: 3 (60%); median age: 52	HTLV-1 without HAM/TSP: 2 (40%) HTLV-1 with HAM/TSP: 2 (40%) Asymptomatic HIV/HTLV-1 1 (20%)	RAL (400 mg bid)	There was a transient reduction in PVL in the two symptomatic patients. No clinical improvement was observed.
Abad-Fernandez et al., 2014 [18]	Cohort, Spain	RAL group: N = 4; male: 4 (26.6%); median age: 51 (48–54). Control group: N = 11(73.4), median age: 50 (46–56).	HIV/HTLV-2: 15 (100%)	Intervention: RAL-based ART (400 mg bid) Control: ART without RAL	There was an initial increase followed by a reduction in PVL in the RAL group. This was not observed in the control group. No changes in CD4+ and CD8+ cell count were detected, regardless of group.
Enose-Akahata et al., 2021 [19]	Clinical trial, USA	RAL group: N = 16 (28.6%); female: 10 (62.5%); mean age: 53.5 Control group: HAM/TSP: N = 13 (23.2%) People without infection: N = 27 (48.2), age and gender not described	HTLV-1 with HAM/TSP:29 (51.8%) People without infection: 27 (48.2%)	Intervention: ART with RAL (400 mg bid) Control group: without ART	There was a subjective improvement in symptoms with the use of RAL, but not in objective clinical measurements. PVL in CSF and PBMC remained stable throughout this study. There were reductions in CD4+ and CD8+ cells in peripheral blood following the use of RAL.

## Data Availability

The raw data supporting the conclusions of this article will be made available by the authors on request.
